# Urinary Bisphenol A Levels Are Associated with Vascular Injury in Mexican Patients with Coronary Artery Disease

**DOI:** 10.3390/ijms27209295

**Published:** 2026-10-20

**Authors:** Fernando Manuel Guerrero-Meza, Marco Antonio Alcocer-Gamba, Juan Pablo Núñez-Urquiza, Rafael Vera-Urquiza, Aquiles Montalvo-Ramos, Santiaga Marisela Ahumada-Solórzano, Haydé Azeneth Vergara-Castañeda, Juan Carlos Solís-Sáinz, Ana Gabriela Hernández-Puga

**Affiliations:** 1 Centro de Investigación Biomédica Avanzada, Facultad de Medicina, Universidad Autónoma de Querétaro, Santiago de Querétaro 76140, Mexicomarco.alcocerg@gmail.com (M.A.A.-G.);; 2 Centro de Estudios Clínicos de Querétaro, Santiago de Querétaro 76000, Mexico; 3 Instituto de Corazón de Querétaro, Santiago de Querétaro 76000, Mexico; 4 Facultad de Ciencias Naturales, Universidad Autónoma de Querétaro, Santiago de Querétaro 76230, Mexico

**Keywords:** bisphenol A, coronary artery disease, atherosclerosis, matrix metalloproteinases, SYNTAX score

## Abstract

Coronary artery disease (CAD) is a leading cause of death worldwide and is associated with standard modifiable cardiovascular risk factors (SMuRFs); however, additional factors may contribute to its development. Bisphenol A (BPA), an endocrine-disrupting chemical has been associated with cardiovascular alterations. We investigated BPA’s possible association with classic risk factors and vascular injury biomarkers in Mexican patients with CAD. Adult patients underwent quantitative coronary angiography and SYNTAX score assessment and were classified as having obstructive (OD) or non-obstructive (NOD) disease. SMuRFs, lipid profile, VCAM-1, MMP-2, MMP-9, and uBPA were determined. Data were analyzed using correlation analysis, Welch’s *t*-test, ANOVA, and adjusted multiple linear regression. Eighty-nine patients aged 30–85 years were evaluated. OD patients had higher uBPA levels (3.46 ± 2.04 ng/mL) than NOD patients (1.25 ± 1.08 ng/mL), and uBPA levels differed according to the number of diseased arteries and diseased artery segments. uBPA was positively associated with MMP-2, MMP-9, SYNTAX score, percentage of stenosis, number of diseased arteries, diseased artery segments, and SMuRFs, and negatively associated with HDLc. SYNTAX score was associated with MMP-2, MMP-9, VCAM-1 and low HDLc levels. Our findings support uBPA levels are associated with vascular injury in Mexican patients with coronary artery disease.

## 1. Introduction

Coronary artery disease (CAD) is a major cause of mortality and disability, and it is the leading cause of death in the world, with approximately 17.8 million deaths each year [1,2]. It is characterized by the presence of atherosclerotic plaques in the coronary arteries, triggered by inflammatory signals such as VCAM-1, MMP-2 and MMP-9, which are released by macrophages, endothelial cells and vascular smooth muscle cells (VSMCs). These signals induce extracellular matrix remodeling within the intima layer through the degradation of collagen and elastin fibers [3,4].

The traditional risk factors associated with CAD are the standard modifiable coronary risk factors (SMuRFs): hyperlipidemia, hypertension, diabetes mellitus and smoking [5]. However, the current presence of patients without traditional risk factors (SMURF-less) has been increasing, supporting the notion that additional factors contribute to vascular damage and the development of coronary heart disease [6]. Cardiovascular toxicants are xenobiotics capable of altering several cellular pathways related to vascular damage. These alterations include the accumulation of fatty deposits in the arteries; the promotion of oxidative, inflammatory, and vascular remodeling processes; endothelial and muscular dysfunction; and the formation of necrotic foci in fatty plaques, leading to cell death [7].

Bisphenol A (BPA) is a chemical used to provide strength and flexibility to plastic products. It is found in everyday products such as aluminum cans, plastic bottles, food and beverage containers, toys, medical devices and thermal paper receipts [8]. BPA can be released from polycarbonate- and PVC-based packaging when exposed to heat or acidic conditions [9,10]. Once leached into food, it is ingested orally and absorbed in the intestine through the ABCC1/MRP1 and MRP3 transport proteins [11]. In 2024, global BPA production was estimated at 6.4 to 8.18 million tons, with projections indicating an increase to 9.3 to 11.23 million tons by 2030 [12,13]. Studies have documented the presence of BPA in environmental samples such as dust (402–3420 ng/g) and surface waters (10–425 ng/L) [14,15]. BPA has been reported in human biological samples from several populations, with differences that reflect demographic factors such as population, age and sex [16,17].

Several studies have shown the relation between environmental chemicals and cardiovascular diseases. BPA exposure has been associated with cardiovascular alterations, including oxidative stress, inflammatory processes, endothelial dysfunction and a decrease in high-density lipoprotein cholesterol (HDLc) levels [18,19,20,21]. Furthermore, it reduces nitric oxide bioavailability, inducing hypertension and endothelial cell necroptosis, while increasing SMC migration to the intima layer and altering MMP-2, MMP-9 and VCAM-1 levels in VSMCs [22,23,24,25], while studies in humans have associated BPA exposure to dyslipidemia development [20,26].

Mexico experiences significant environmental exposure to BPA [27,28] and a high prevalence of CAD. To date, there is insufficient evidence regarding the association between BPA and vascular injury in the Mexican population. The objective of this study was to conduct an initial exploratory assessment of BPA presence in the Mexican population and to examine its possible association with classic risk factors, vascular injury biomarkers and SYNTAX score. We identified higher urinary BPA (uBPA) levels in patients with obstructive disease, as well as higher number of diseased arteries and diseased artery segments. Furthermore, uBPA levels were positively associated with MMP-2, MMP-9, non-HDLc, SYNTAX score, percentage of stenosis, number of diseased arteries, diseased artery segments and the number of SMuRFs, while being negatively associated with HDLc levels, suggesting that BPA exposure may be related to vascular injury in patients with CAD.

## 2. Results

### 2.1. Characteristics of the Population

Eighty-nine patients with CAD were recruited, 88% were men and 12% women. The mean age was 63.1 ± 11 years among men and 66.2 ± 10 years among women. Male patients were classified as normal weight (57%) or overweight (43%); female patients were classified as normal weight (83%) or overweight (17%). The prevalence of SMuRFs in the population was as follows: hypertension (52%), dyslipidemia (92%), diabetes mellitus (34%) and smoking (49%). Among the patients, 80% had more than one SMuRF and only 1% had no SMuRFs. The most frequent combination of SMuRFs was hypertension and dyslipidemia, present in 47% of the total population (Table 1).

ACS was diagnosed in 75% of the patients, compared to 25% with CCD. Among ACS patients, 45% had STEMI, 36% had NSTEMI and 19% had unstable angina. OD was observed in 78 patients (88%), while 11 patients (12%) had NOD. Based on the SYNTAX score, 47 patients were classified as low risk, 21 as intermediate risk and 10 as high risk. Eleven patients had a SYNTAX score of zero, either due to non-significant lesions (<50% stenosis, *n* = 6) or the absence of detectable plaques (*n* = 5) (Table 1).

**Figure 1:**
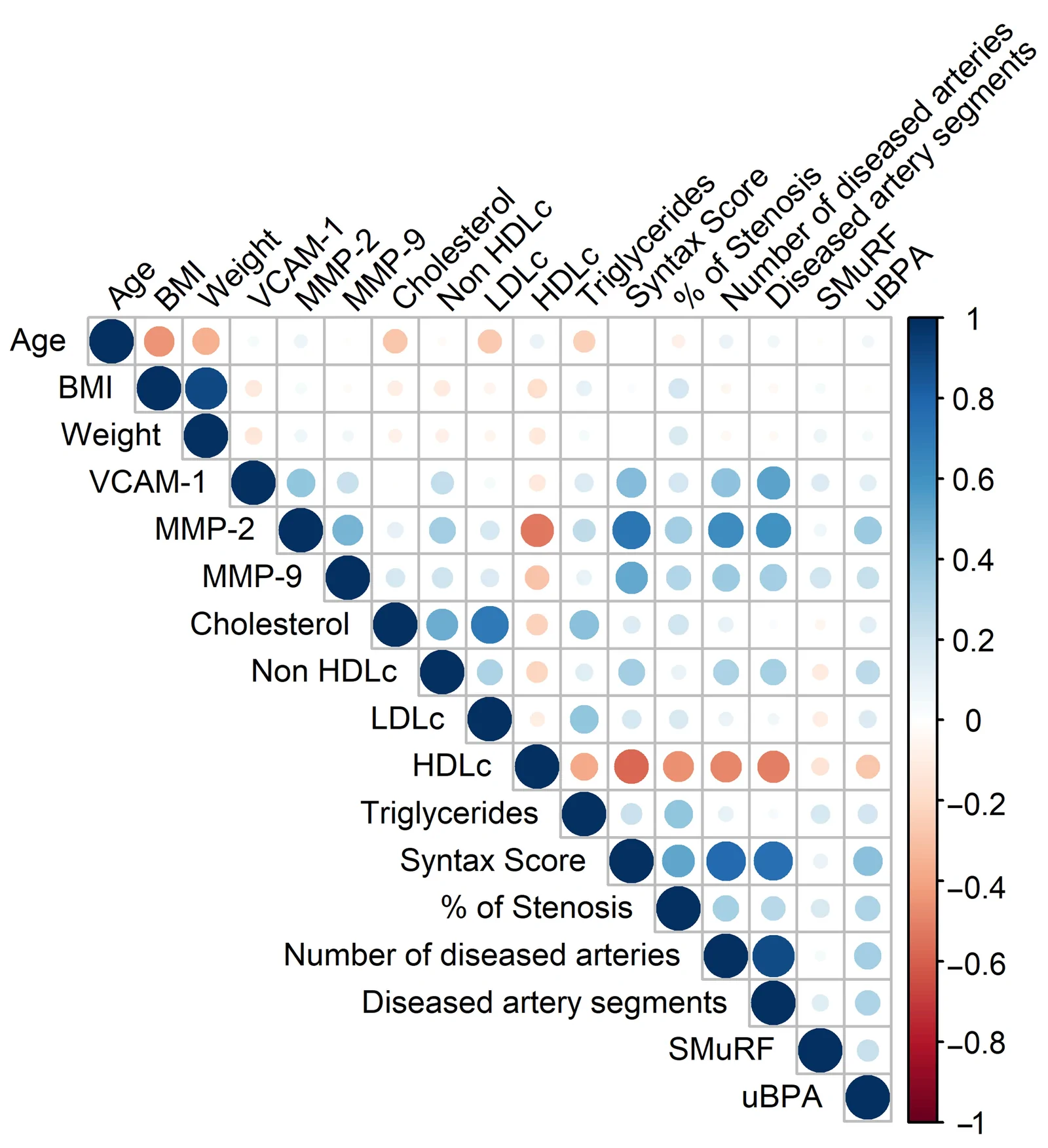
Correlation matrix showing the associations between vascular-damage variables and uBPA levels in the population sample. Spearman correlation analyses were conducted among the variables of interest. Spearman’s correlation coefficients (r_s_) are represented by circles. Circle size is proportional to the absolute magnitude of the r_s_, whereas circle color indicates the direction of the association. Positive correlation values are represented in a blue gradient and negative correlation values are represented in a red gradient. *n* = 89, *p* < 0.05.

The correlation matrix showed that uBPA levels had a positive significant correlation with MMP-2 (r_s_ = 0.367), MMP-9 (r_s_ = 0.232), SYNTAX score (r_s_ = 0.415), percentage of stenosis (r_s_ = 0.306), non-HDLc (r_s_ = 0.266), number of diseased arteries (r_s_ = 0.349), number of diseased artery segments (r_s_ = 0.307) and number of SMuRFs (r_s_ = 0.228). Conversely, a negative correlation was observed between uBPA and HDLc (r_s_ = −0.274). The analysis also revealed a positive correlation between the percentage of stenosis and levels of tryglicerides (r_s_ = 0.390) and a negative correlation with HDLc (r_s_ = −0.460). The SYNTAX score showed a positive correlation with non-HDLc (r_s_ = 0.344) and a negative correlation with HDLc (r_s_ = −0.579) (Figure 1).

**Figure 2:**
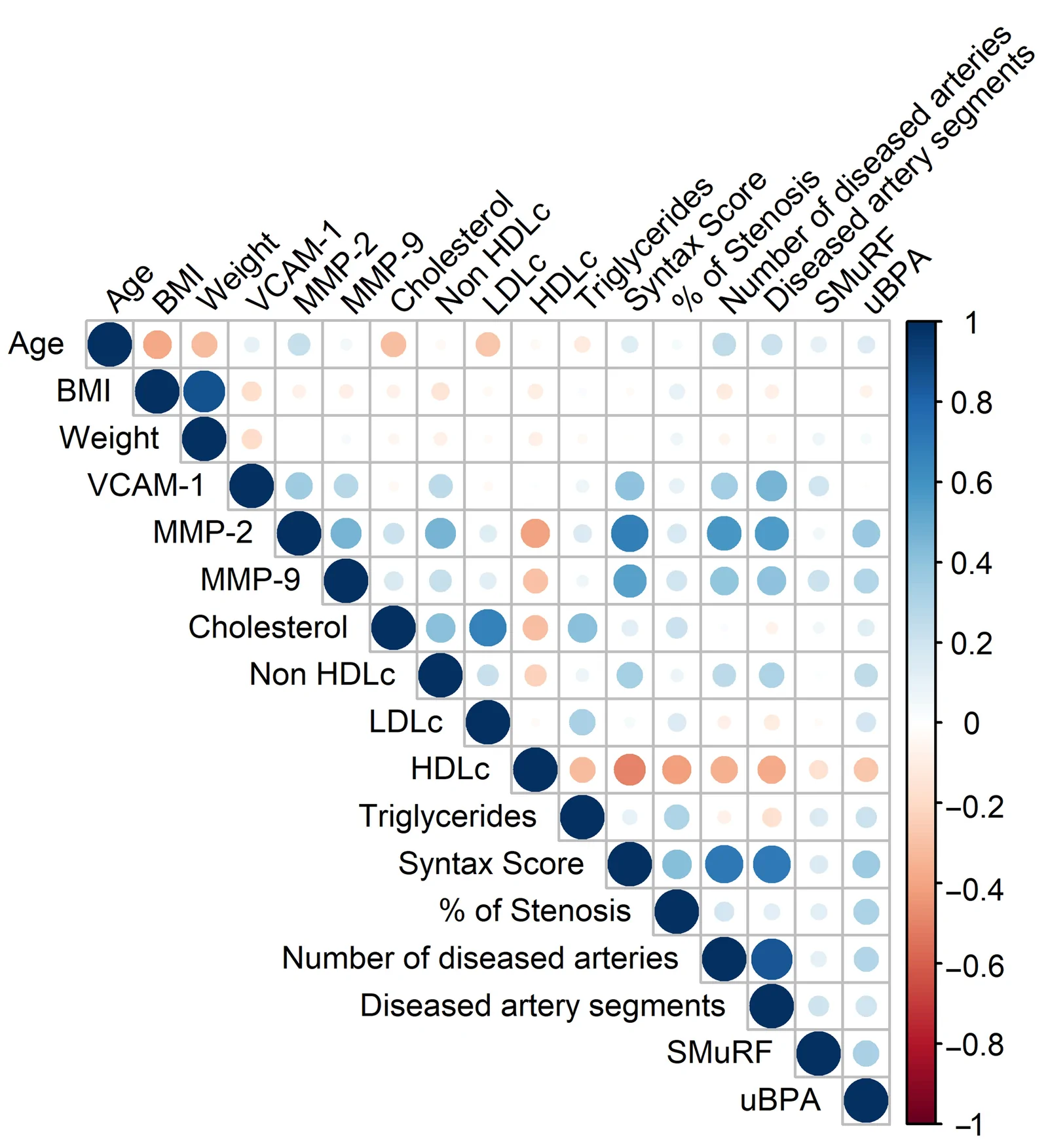
Correlation matrix showing the associations between vascular-damage variables and uBPA levels in patients with ACS. Spearman correlation analyses were conducted amongst the variables of interest. Spearman’s correlation coefficients (r_s_) are represented by circles. Circle size is proportional to the absolute magnitude of the r_s_, whereas circle color indicates the direction of the association. Positive correlation values are represented in a blue gradient and negative correlation values are represented in a red gradient. *n* = 67, *p* < 0.05.

Additionally, subgroup-specific correlation matrices were generated for patients with ACS and CCD, revealing different patterns of association within each clinical subgroup. In the ACS group, a significant correlation was observed between uBPA and vascular-damage variables: MMP-2 (r_s_ = 0.372), MMP-9 (r_s_ = 0.296), SYNTAX score (r_s_ = 0.366), percentage of stenosis (r_s_ = 0.319), number of diseased arteries (r_s_ = 0.292), presence of SMuRFs (r_s_ = 0.328) and HDLc (r_s_ = −0.280). Significant correlations were also determined between SYNTAX score and VCAM-1 (r_s_ = 0.408), MMP-2 (r_s_ = 0.684), MMP-9 (r_s_ = 0.544) and HDLc (r_s_ = −0.497) (Figure 2).

**Figure 3:**
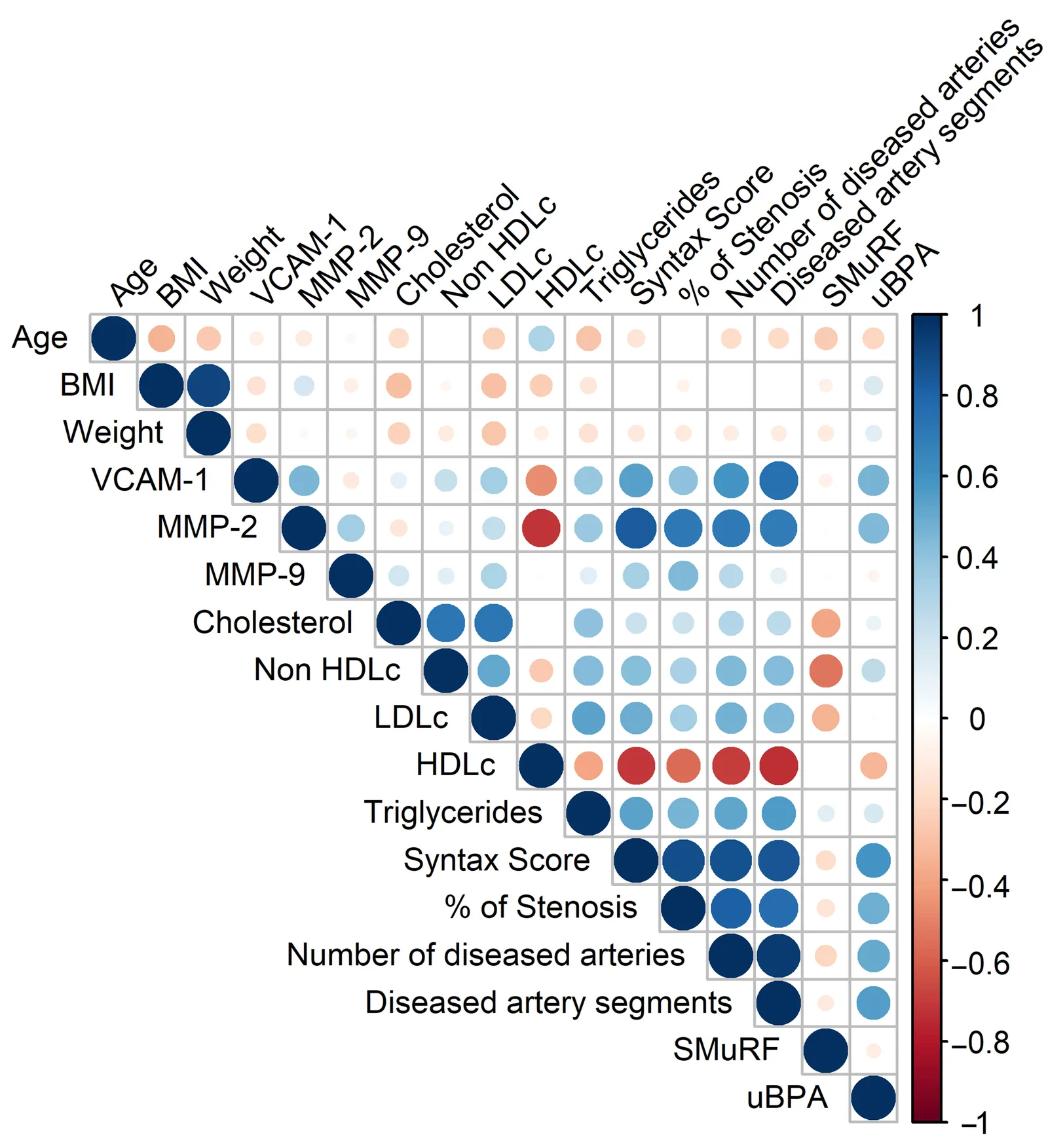
Correlation matrix showing the associations between vascular-damage variables and uBPA levels in patients with CCD. Spearman correlation analyses were conducted amongst the variables of interest. Spearman’s correlation coefficients (r_s_) are represented by circles. Circle size is proportional to the absolute magnitude of the r_s_, whereas circle color indicates the direction of the association. Positive correlation values are represented in a blue gradient and negative correlation values are represented in a red gradient. *n* = 22, *p* < 0.05.

In the CCD group, a positive correlation was found between uBPA levels and VCAM-1 (r_s_ = 0.460), MMP-2 (r_s_ = 0.443), SYNTAX score (r_s_ = 0.593), percentage of stenosis (r_s_ = 0.480), number of diseased arteries (r_s_ = 0.501) and number of diseased segments (r_s_ = 0.558), while positive relationships were also found between SYNTAX score and VCAM-1 (r_s_ = 0.548), MMP-2 levels (r_s_ = 0.835), and TG (r_s_ = 0.530), and a negative correlation with HDLc (r_s_ = −0.703) (Figure 3).

### 2.2. Vascular-Damage Biomolecule Levels

**Figure 4:**
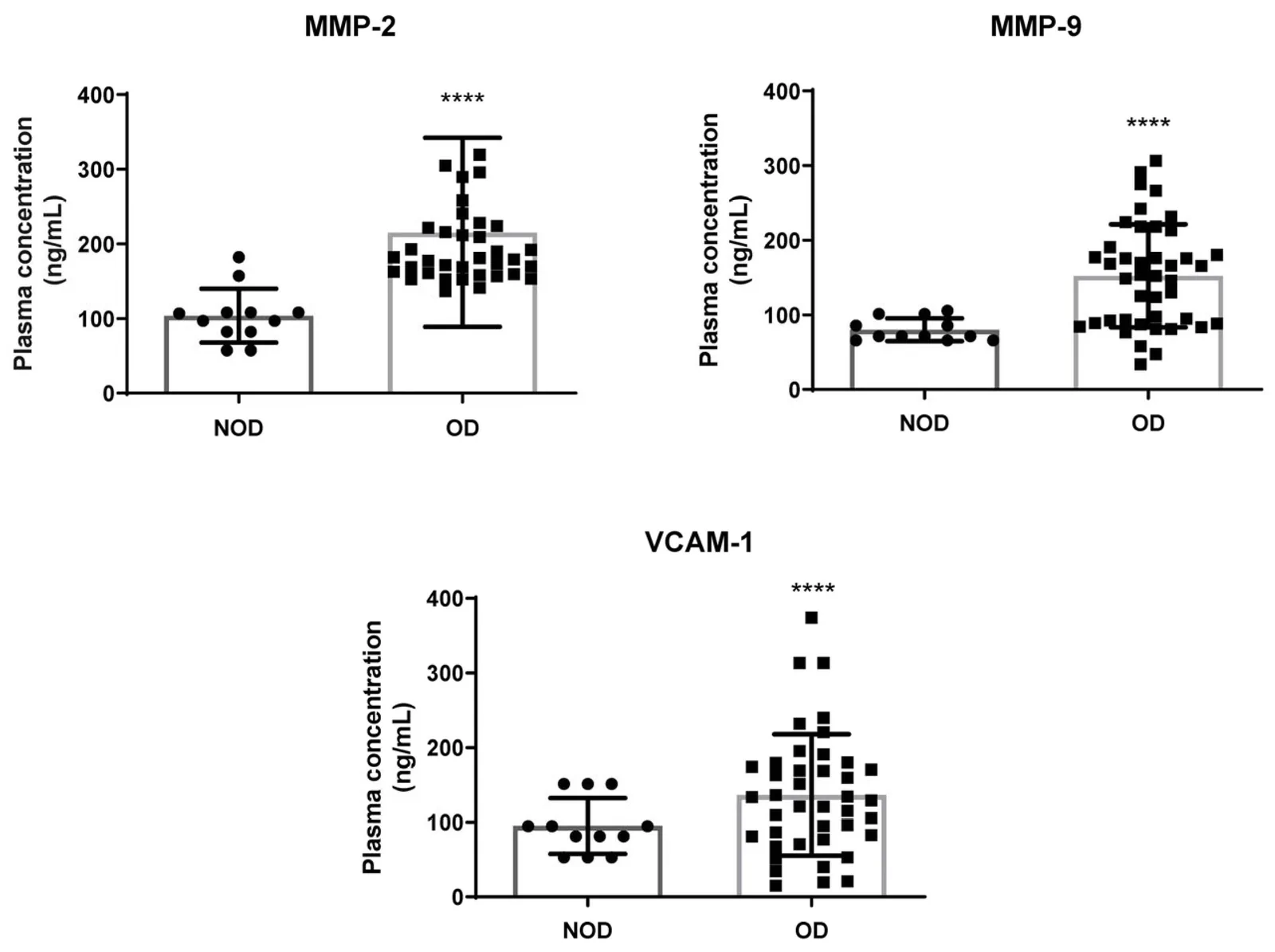
Comparison of vascular-damage marker levels between NOD and OD groups. Data are presented as mean ± SD. Differences between groups were determined using Welch’s two sample *t*-tests. A *p*-value < 0.05 was considered statistically significant. *n* = 89, **** *p* ≤ 0.003.

The levels of vascular-damage-associated biomolecules VCAM-1, MMP-2 and MMP-9 were significantly higher in patients with OD compared to those with NOD (Figure 4). Furthermore, positive associations were found between SYNTAX score and the biomolecules VCAM-1 (r_s_ = 0.433), MMP-2 (r_s_ = 0.726) and MMP-9 (r_s_ = 0.514), with the strongest correlation observed for MMP-2 (*p* = 8.64 × 10^−16^), while weaker but significant correlations with arterial stenosis severity [MMP-2 (r_s_ = 0.349) and MMP-9 (r_s_ = 0.295)] and number of damaged arteries were also observed [VCAM-1 (r_s_ = 0.407), MMP-2 (r_s_ = 0.620) and MMP-9 (r_s_ = 0.367)], suggesting a relationship of the molecules with increased coronary-mortality risk (Figure 1).

### 2.3. Urinary BPA Levels

Urine samples showed absolute uBPA concentrations ranging from 0.3 to 37.12 ng/mL, with a mean value of 4.45 ± 6.4 ng/mL. When stratified by sex, male patients exhibited higher uBPA concentrations than female patients. Notably, four individuals from the overall cohort exhibited markedly elevated uBPA levels ranging from 21.2 to 37.12 ng/mL (Table 2). These data were excluded for further analyses in order to maintain the sample’s dispersion at minimum (Me = 2.59; C.V. = 65.53%, A = 1.230, K = 1.234). Sensitivity analyses performed including and excluding these measurements yielded consistent further inferential results.

**Figure 5:**
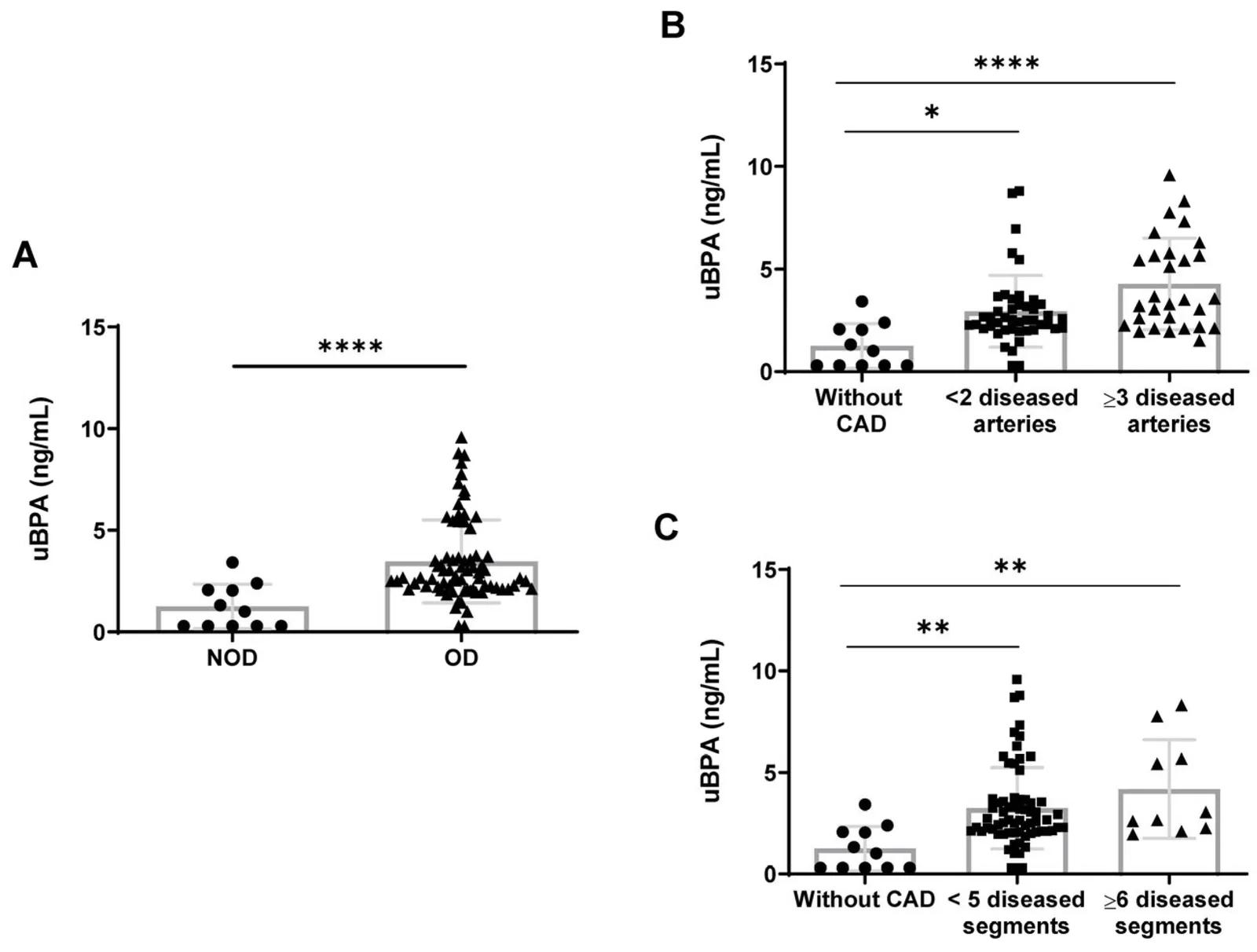
Comparison of uBPA levels between NOD and OD groups. Data are presented as mean ± SD. The data show uBPA levels in: (**A**) NOD and OD groups, (**B**) patients categorized by the number of diseased arteries, and (**C**) patients categorized by the number of diseased arterial segments. To evaluate differences between groups, data were analyzed using Welch’s *t*-test (Figure 5A) or one-way ANOVA followed by Tukey’s post hoc multiple comparison test (Figure 5B,C). A *p*-value < 0.05 was considered statistically significant. *n* = 85, * *p* < 0.03, ** *p* < 0.002, **** *p* < 0.0001.

Patients with OD exhibited higher mean uBPA levels (3.47 ± 2.04 ng/mL) compared to those with NOD (1.25 ± 1.09 ng/mL) (Figure 5A). The glomerular filtration rate for NOD and OD groups were 80.6 ± 13.1 and 75.1 ± 10.2 mL/min/1.73m^2^, respectively; no significant difference between the groups was found. As shown in Figure 5B, uBPA concentrations were significantly higher in groups with a greater number of coronary arteries affected by atherosclerotic lesions (*p* < 0.05) compared to the NOD group. Furthermore, a significant increase in uBPA levels was also observed in patients with a greater number of affected arterial segments compared to the NOD group (*p* = 0.0005) (Figure 5C).

### 2.4. Model Analysis

To further explore the relationship of uBPA levels and vascular-damage-associated biomolecules with SYNTAX score, a multiple linear regression model was constructed using the SYNTAX score as the dependent variable and was adjusted considering confounding factors: age, sex, BMI, smoking, hypertension, diabetes, LDLc and triglycerides. In the first model, MMP-2, MMP-9, VCAM-1 and HDLc were identified as significant predictors, but not BPA (R^2^ = 0.643, *p* = 3.76 × 10^−12^):

(1) SYNTAX score = 21.04 + (0.070 × MMP-2) + (0.034 × MMP-9) + (0.024 × VCAM-1) − (0.287 × HDLc)

A second model was calculated to evaluate the relationship between uBPA levels and vascular-damaged-associated biomolecules and the number of arterial segments; a significant correlation was only observed between the number of arterial segments with significant lesions and MMP-2, HDLc, TG and VCAM-1 (R^2^ = 0.574, *p* = 5.54 × 10^−10^):

(2) Number of diseased artery segments = 4.53 + (0.008 × MMP-2) − (0.067 × HDLc) + (0.009 × VCAM-1) − (0.008 × TG)

A 2 × 2 contingency analysis was performed to evaluate the association between elevated uBPA levels and obstructive coronary disease among patients with CAD. Among the exposed patients (uBPA > 1.0 ng/mL), 76 presented OD and six presented NOD. In contrast, among the unexposed patients (uBPA ≤ 1.0 ng/mL), two had OD and five had NOD. The relative risk (RR) of OD associated with uBPA > 1.0 ng/mL was 3.24 (95% CI: 1.004–10.48; *p* = 0.0002), indicating that the proportion of patients with OD was approximately 3.24-fold higher among patients with elevated uBPA levels.

## 3. Discussion

The present study provides novel insights into BPA levels in Mexican patients diagnosed with CAD. Our analysis integrates clinical, biochemical and molecular data, shedding light on how environmental exposure to endocrine-disrupting chemicals like BPA is associated with biomarkers of vascular injury and traditional cardiovascular risk factors of CAD.

The studied population showed differences by sex, age, SMuRFs, vascular damage and uBPA concentrations. Biological differences between sexes—including hormonal, genetic and cellular factors—contribute to variations in disease presentation [29]. Dyslipidemia was the most prevalent SMuRF present in 91% of the patients. Elevated LDLc and non-HDLc levels, combined with reduced HDLc, are major contributors to the development and progression of atherosclerotic plaque. These lipid abnormalities increase the risk of vascular obstruction and the morbidity and mortality associated with CAD in individuals over 20 years of age, with risk rising progressively across older age groups [30]. Atherosclerotic plaques have been documented in both young and elderly patients in the Mexican population, with a trend toward increasing plaque burden and severity over time [1].

Alterations in LDLc, non-HDLc and HDLc may be induced by different mechanisms. For example, expression of ATP-binding cassette transporters A1 and G1 (ABCA1/G1) is downregulated in macrophages and vascular smooth muscle cells, which reduces cholesterol efflux to HDLc particles and impairs reverse cholesterol transport [31]. This may explain why HDLc values alone do not always reflect a protective lipid profile, since HDLc is known for its anti-inflammatory and atheroprotective properties, including inhibition of LDLc oxidation and promotion of endothelial function and reverse cholesterol transport [32].

Dyslipidemia and hypertension are prevalent in the Mexican population. The presence of CAD in normal-weight individuals with low HDLc and elevated non-HDLc suggests a metabolic disruption possibly linked to environmental exposure rather than lifestyle alone [30]. Over recent years, the prevalence of early atherosclerosis and fatty streaks has increased in the Mexican population. This rise is attributable to globalization, the adoption of unhealthy lifestyle habits and the widespread presence of SMuRFs, all of which contribute to the growing burden of CAD in Mexico and across Latin America [33].

Results showed uBPA levels were higher in men than in women. This difference may be explained by variations in environmental exposure, dietary habits, temporal exposure and differences in BPA metabolism. Mahalingaiah et al. (2008) reported that women exhibited 9% lower BPA concentrations than men, supporting the existence of sex-related differences in internal BPA exposure [34]. In addition, endocrine mechanisms have been proposed as potential contributors. Takeuchi and Tsutsumi (2002) reported a positive correlation between circulating BPA concentrations and androgen levels in healthy men and women with polycystic ovary syndrome, whereas healthy women exhibited lower BPA concentrations [35]. Likewise, sex-related differences in the metabolic pathways responsible for BPA detoxification, particularly glucuronidation and sulfation, may influence BPA clearance and contribute to differences in internal BPA burden [36]. Experimental and epidemiological studies suggest that BPA may promote adipose tissue accumulation and alter metabolism and body composition. This condition, together with differences between men and women in fat distribution, hormone regulation and xenobiotic metabolism, may make men more susceptible to the effects of BPA and contribute to cardiovascular alterations [16].

The NOD and OD groups showed differences in uBPA levels and the extent of CAD. Higher BPA concentrations were observed in patients with OD, a greater number of diseased arteries, and more affected segments, while also demonstrating an association with the SYNTAX score, and non-HDL and HDLc levels. It is known that BPA can impair lipid homeostasis and reduce endothelial nitric oxide availability, promoting vasoconstriction, increased peripheral resistance and elevated blood pressure [22]. BPA can modulate lipid metabolism in LDLR^−/−^ mice, by suppressing hepatic ApoA1 via NF-κB activation, reducing HDLc and increasing total lipid levels [19]. Similar findings have been reported in human cohorts, linking BPA concentration with elevated lipid levels [20,21,37]. BPA can increase reactive oxygen species (ROS), decrease antioxidant enzymes and nitric oxide and promote endothelial dysfunction, leading to higher TNFα and VCAM-1 levels, apoptosis and necrotic core formation in arterial walls [38,39]. ROS such as hydrogen peroxide and peroxynitrite contribute to mitochondrial dysfunction, protein nitration and membrane instability, further enhancing inflammation through IL-1, IL-6 and TNF pathways [40].

Other studies have evaluated the relationship between CAD and BPA. Melzer et al. [41] observed a correlation between severe vascular disease and uBPA concentrations, finding levels of 1.25–69.4 ng/mL. Similarly, Alizadeh-Fanalou et al. [42] found a relationship between BPA and necrotic lesions in patients, as evidenced by elevated CK-MB and troponin levels. Our study found uBPA levels up to 37.5 ng/mL, comparable to Melzer’s report and these were associated with significant arterial damage. Unlike previous studies, we used the SYNTAX score, which allows for better assessment of coronary complexity, severity and risk of death [41,42]. In Mexico, BPA has been detected in surface water (15–30 µg/L in the north; 1–10 ng/L in the center of the country) and in commonly used consumer products such as canned foods and water bottles [43,44]. In biological samples from the Mexican population, mean BPA levels of 35.22 μg/L have been reported [27], consistent with our findings.

Our results showed a linear relationship between uBPA levels and both MMP-2 and MMP-9. BPA exposure has been linked to increased expression of MMP-9 in endothelial cells and to activation of the inflammatory pathway by elevating levels of NFκB, caspases 8 and 9 and TNFα [19,45]. Furthermore, VCAM-1 activates NADPH oxidase, which in turn activates VCAM-1 and MMPs, promoting the presence of ROS [45,46]. Likewise, BPA may modulate MMP expression through activation of the inflammatory response via Toll-like receptor 4 (TLR4), leading to the expression of membrane-type matrix metalloproteinases (MT-MMPs) and subsequent activation of proMMP-13 and MMP-2, resulting in remodeling of the extracellular matrix in the coronary artery layer [47]. Further studies are required to elucidate whether BPA promotes atherosclerotic plaque development through these molecular targets.

The levels of VCAM-1, MMP-2 and MMP-9 were significantly higher in patients with OD compared to those with NOD. Multiple regression models showed MMP-2, MMP-9, VCAM-1 and low HDLc levels were consistently associated with an increased risk of vascular injury and with the number of diseased artery segments. These findings emphasize the inflammatory and remodeling activity of vascular tissue and reinforce their relevance as potential biomarkers of atherosclerotic progression. MMPs and VCAM-1 determination allowed us to analyze patients with chronic lesions who did not present elevated cardiac enzymes but did have active vascular lesions characterized by vascular remodeling and arterial inflammation [4].

Although uBPA was significantly correlated with MMP-2, MMP-9, HDLc, and SYNTAX score, multiple linear regression analysis showed no significant association between uBPA and the SYNTAX score or the number of diseased arterial segments after adjustment for vascular biomarkers and clinical factors. These findings suggest that the association between BPA exposure and CAD may be related to alterations in vascular remodeling and lipid metabolism, as previously reported [19,23]. Since these processes could be involved in the relationship between BPA exposure and vascular damage, their inclusion in the regression models may have reduced the observed association with uBPA. However, we cannot determine whether these relationships are due to indirect biological mechanisms or shared clinical factors. Therefore, further studies are needed to clarify the potential role of BPA in coronary atherosclerosis.

Additionally, we observed a significant association between elevated uBPA levels and obstructive coronary disease among patients with CAD (RR = 3.24). Taken together, our results provide new evidence of BPA exposure in the Mexican population and suggest a potential role for BPA in promoting CAD.

To our knowledge, this is the first study in Mexico to evaluate the cardiovascular association of BPA at this level of clinical detail. One of the key strengths of our study is the clinical diversity of the population. The OD group included patients with acute myocardial infarction and unstable angina, whereas the NOD group comprised patients with chronic coronary disease and atherosclerotic lesions showing less than 50% stenosis. This clinical diversity allows the analysis of CAD from a broader perspective, integrating anatomical obstruction with various clinical scenarios and vascular-damage profiles that heighten the risk of fatal myocardial infarction, and provide a more comprehensive understanding of the cellular mechanisms associated with CAD progression.

Several limitations of this study should be acknowledged. First, given its descriptive nature, we had limited ability to control for diverse environmental exposure and daily stimuli, which represents an inherent challenge in human research. Second, as an initial exploratory assessment, the relatively small sample size may have restricted the precision of our statistical estimates. Therefore, future large-scale investigations are warranted to validate these findings.

Additionally, a single uBPA measurement only reflects recent exposure due to the short half-life of the compound. Prospective cohort studies are needed to better characterize the magnitude and duration of BPA exposure and to clarify its role in CAD development. Finally, pre-interventional uBPA measurements or blood sample determinations could provide a more comprehensive assessment of exposure. Future studies should also normalize uBPA data using urinary creatinine levels or specific gravity to improve the understanding of how BPA influences the presence and severity of CAD.

## 4. Materials and Methods

### 4.1. Study Population

Eighty-nine men and women aged 30–85 years admitted to the Hemodynamic Department at the Heart Institute of Querétaro, Mexico, between March and November 2023 were included. Inclusion criteria were age over 30 years, Mexican nationality, diagnosis of CAD and an evaluation by coronary angiography. Exclusion criteria were autoimmune diseases (lupus erythematosus, Sjögren’s disease, Addison’s disease, Hashimoto’s disease, multiple sclerosis) and/or metastatic cancer (except for those in remission for more than 5 years). Participation in the study was voluntary; patients signed informed consent and could withdraw at any time.

### 4.2. Clinical Data

Patients with a medical indication for coronary angiography and a diagnosis of acute coronary syndrome (ACS) without ST elevation (NSTEMI), with ST elevation (STEMI), unstable angina, or chronic coronary disease (CCD) were recruited. All patients agreed to participate and signed informed consent. Patient information was collected from clinical records, including risk factors such as sex, age, body mass index (BMI) and lipid profile. The lipid profile included blood levels of TC, non-HDLc, LDLc, HDLc and triglycerides (TGs). All information was less than four weeks old. Comorbidities were identified according to SMuRFs: hypertension (blood pressure > 140/90 mm Hg), diabetes (fasting glucose > 126 mg/dL, A1c > 6.5%), dyslipidemia (TC > 200 mg/dL, TG > 150 mg/dL, LDLc > 55 mg/dL, non-HDLc > 85 mg/dL, HDLc < 60 mg/dL) and smoking. Patients did not suspend their pharmacological treatment during the study.

### 4.3. Quantitative Coronary Angiography

Coronary angiography was performed by the interventional cardiology staff of the Heart Institute. The coronary catheterization report was used to record the conclusions of the interventional cardiologist. Quantitative coronary angiography (QCA) was performed using Allura Xper FD10 722003 (Philips Healthcare, Netherlands) and SyncVision software version v3.x (Philips Healthcare, Netherlands) to quantify the percentage of stenosis, atherosclerotic lesion area and the presence of other lesions along the blood vessel.

After the procedure, the patients received intravenous hydration for 12 h to promote excretion of the contrast medium used in the angiography (iopromide). Subsequently, the patient continued to be monitored (approximately 12–24 h) without intravenous hydration and received only water and food orally. The hydration solution was used to maintain a patent IV line, where 3–5 mL of physiological saline solution was administered every 8 h (intermittent flushing) to maintain venous access, in accordance with the Protocol for the Standardized Management of Patients with Peripheral Catheters. Patients were admitted in the hospital in a period of 24–36 h.

Patients were classified as having non-obstructive disease (NOD) or obstructive disease (OD). OD was defined as >50% stenosis of the vessel lumen in an artery with a diameter >1.5 mm. NOD included patients with plaques causing <50% stenosis. Participants in the NOD group of the study, when evaluated in the emergency department or at their initial consultation, met diagnostic criteria for complications of CAD (acute myocardial infarction, unstable angina, chronic coronary artery disease, plaque on CT angiography, ventricular hypokinesis on ultrasound). After intervention, these patients were found to have myocardial muscle bridging, slow blood flow, and a positive stress test. Cardiovascular risk was calculated using the SYNTAX score according to the official SYNTAX score website (https://syntaxscore.org/), which considers factors such as dominance, the artery and segment with the lesion, type of occlusion, bifurcation, type of injury, tortuosity, calcification, and diffuse disease, among others. The calculation was carried out by a team of three health professionals. Patients were classified by risk of cardiovascular mortality as low risk (<22 points), intermediate risk (23–32 points), or high risk (>33 points) according to the American Heart Association classification.

### 4.4. Analysis of Vascular-Damage Markers

Arterial blood samples were collected from patients before the angiographic procedure. Samples were collected in tubes and centrifuged at 3000 rpm for 15 min to separate serum from the red blood cell fraction. Serum concentrations of MMP-2, MMP-9 and VCAM-1 were determined using an ELISA immunoassay (Sigma-Aldrich, St. Louis, MO, USA#RAB0365, #RAB0372 and #RAB0505, respectively). A first-morning single urine sample was collected 24 h after angiography, centrifuged at 3000 rpm for 10 min and the supernatant was collected and stored. All samples were stored in BPA-free sterile tubes or containers at −20 °C until analysis.

### 4.5. Quantification of Bisphenol A in Urine Samples

Absolute urinary BPA levels estimates were determined by ELISA immunoassay using the MBS269957 kit according to the supplier’s instructions and reported in ng/mL (MyBioSource, San Diego, CA, USA) [48]. uBPA concentrations were reported as absolute values and were not adjusted for urinary creatinine or specific gravity.

### 4.6. Statistical Analysis

Variables normality distribution was assessed with the Kolmogorov–Smirnov test. Descriptive statistics were expressed as mean and standard deviation or as frequencies and percentages. A simple correlation matrix was constructed to examine relationships between study variables. Welch’s *t*-test was used to compare MMPs, VCAM-1 and uBPA levels in patients with NOD and OD. A one-way ANOVA was performed, followed by Tukey’s post hoc multiple comparison test, to evaluate differences in uBPA levels in patients according to the number of diseased arteries and the number of diseased arterial segments. Multiple linear regression analyses were performed to evaluate the independent association of uBPA with CAD. The SYNTAX score and the number of diseased artery segments were analyzed as dependent variables in separate models. The SYNTAX score model included uBPA, MMP-2, MMP-9, VCAM-1, and HDLc, whereas the model for the number of diseased arterial segments included uBPA, MMP-2, VCAM-1, and HDLc. Both models were additionally adjusted for clinically relevant potential confounders, including age, sex, BMI, smoking, hypertension, diabetes, LDLc, and triglycerides. All prespecified predictors were entered simultaneously using the enter method.

Fisher’s exact test was used to assess the association between elevated uBPA levels and OD versus NOD among patients with CAD. Patients were classified according to uBPA levels as exposed (uBPA > 1.0 ng/mL) or unexposed (uBPA ≤ 1.0 ng/mL). The cutoff of uBPA > 1.0 ng/mL was established based on the minimal concentrations of urinary BPA reported in human populations [27,42]. The relative risk (RR) was calculated as the proportion of patients with OD in the exposed group divided by the corresponding proportion in the unexposed group. Statistical significance was set at *p* <0.05. Analyses were performed using GraphPad Prism version 8.4.3 and IBM^®^ SPSS Statistics version 27.0.1.0.

## 5. Conclusions

The high prevalence of CAD in Mexico represents a critical public health challenge that may be exacerbated by widespread exposure to environmental contaminants, including BPA. Our findings demonstrated that uBPA levels are associated with traditional cardiovascular risk factors, SYNTAX score and vascular injury markers, specifically MMP-2 and MMP-9, which are involved in extracellular matrix remodeling. Furthermore, elevated uBPA levels were found in CAD patients with a greater number of diseased arteries and more affected arterial segments. Further prospective and mechanistic studies are warranted to determine whether BPA exposure contributes to the development or progression of CAD.

## Figures and Tables

**Table 1:** Characteristics of the study population.

Characteristic	Value (s)	Mean ± SD
Total population	89	
Male	88%	
Female	12%	
Age		
Male	35–85	63.1 ± 11.5
Female	47–80	66.1 ± 10.4
BMI (kg/m^2^)	19.1–47.9	24.5 ± 3.9
Male	57% (<24.9)	25 ± 3.7
Female	83% (<24.9)	21.7 ± 3.9
**Lipids (mg/dL)**		
Total Cholesterol	74–284	187.9 ± 58.5
Non-HDLc	50–249	148.1 ± 58.5
LDLc	26–196	122.1 ± 45.5
HDLc	20–81	36.8 ± 10.3
Triglycerides	68–384	159.6 ± 74.1
**SMuRF**		
Hypertension	52%	
Dyslipidemia	92%	
Diabetes Mellitus	34%	
Smoking	49%	
**Cardiovascular Disease**		
Acute Coronary Syndrome	75%	
STEMI	45%	
NSTEMI	36%	
Unstable Angina	19%	
Chronic Coronary Disease	25%	
**QCA Classification**		
Obstructive Disease	88%	
Non-Obstructive Disease	12%	
**SYNTAX Score**		
Low	47	
Intermediate	21	
High	10	
Zero	11	

**Table 2:** uBPA concentrations in groups stratified by sex.

Study Group	NOD	OD	
	Range (Mean ± SD)		*p*-Value
Women	0.3–2.39 (0.99 ± 1.2)	1.95–6.97 (3.36 ± 1.7)	0.046
Men	0.3–3.42 (1.34 ± 1.11)	0.3–37.12 (5.10 ± 7.09)	0.0003

Data is shown in ng/mL. Differences between groups were determined using two-tailed Welch’s *t*-tests. A *p*-value < 0.05 was considered statistically significant. *n* = 85.

## Data Availability

The original contributions presented in this study are included in the article. Further inquiries can be directed to the corresponding authors.

## Abbreviations

The following abbreviations are used in this manuscript:

ACS	Acute coronary syndrome
BPA	Bisphenol A
CAD	Coronary artery disease
CCD	Chronic coronary disease
ELISA	Enzyme-Linked Immunosorbent Assay
HDLc	High-density lipoprotein cholesterol
LDLc	Low-density lipoprotein cholesterol
MMPs	Matrix metalloproteinases
NOD	Non-obstructive disease
Non-HDLc	Non-HDL cholesterol
OD	Obstructive disease
PVC	Polyvinyl chloride
SMURFs	Standard modifiable coronary risk factors
SYNTAX	Synergy Between Percutaneous Coronary Intervention with TAXUS and Cardiac Surgery
TC	Total cholesterol
VCAM-1	Vascular Cell Adhesion Molecule 1
VSMC	Vascular smooth muscle cell
